# Diazonium Chemistry for the Bio-Functionalization of Glassy Nanostring Resonator Arrays

**DOI:** 10.3390/s150818724

**Published:** 2015-07-30

**Authors:** Wei Zheng, Rongbing Du, Yong Cao, Mohammad A. Mohammad, Steven K. Dew, Mark T. McDermott, Stephane Evoy

**Affiliations:** 1 Department of Electrical and Computer Engineering, University of Alberta, Edmonton, AB T6G 2V4, Canada; E-Mails: wzheng2@ualberta.ca (W.Z.); mam20@ualberta.ca (M.A.M.); sdew@ualberta.ca (S.K.D.); 2 Department of Chemistry and National Institute for Nanotechnology, University of Alberta, Edmonton, AB T6G 2G2, Canada; E-Mails: rdu@ualberta.ca (R.D.); yong2@ualberta.ca (Y.C.); mark.mcdermott@ualberta.ca (M.T.M.)

**Keywords:** biosensors, nanomechanics, nanostrings, interferometry

## Abstract

Resonant glassy nanostrings have been employed for the detection of biomolecules. These devices offer high sensitivity and amenability to large array integration and multiplexed assays. Such a concept has however been impaired by the lack of stable and biocompatible linker chemistries. Diazonium salt reduction-induced aryl grafting is an aqueous-based process providing strong chemical adhesion. In this work, diazonium-based linker chemistry was performed for the first time on glassy nanostrings, which enabled the bio-functionalization of such devices. Large arrays of nanostrings with ultra-narrow widths down to 10 nm were fabricated employing electron beam lithography. Diazonium modification was first developed on SiCN surfaces and validated by X-ray photoelectron spectroscopy. Similarly modified nanostrings were then covalently functionalized with anti-rabbit IgG as a molecular probe. Specific enumeration of rabbit IgG was successfully performed through observation of downshifts of resonant frequencies. The specificity of this enumeration was confirmed through proper negative control experiments. Helium ion microscopy further verified the successful functionalization of nanostrings.

## 1. Introduction

Biological analysis technologies play an important role in many fields such as disease biomarker diagnosis and monitoring, drug discovery, and molecular analysis [1,2,3,4,5,6,7]. These platforms aim to provide rapid, sensitive and selective recognition of target analytes [6,8,9,10,11]. Established techniques include enzyme-linked immunosorbent assay (ELISA), western blotting, polymerase chain reaction (PCR), and fluorescence conjugated reagents. These assays require labels such as fluorescent dyes and enzymes to properly identify the target. The introduced labels can be toxic to the biological reagents and interfere with the biological or chemical phenomena being investigated. Label-free approaches, on the other hand, keep the biochemical system mostly unperturbed. Compared with the traditional bioassay techniques, label-free detection would offer a more reliable assessment [12,13]. Recent years have seen intensive efforts towards the development of such platforms. Techniques include surface plasmon resonance (SPR), surface-enhanced Raman scattering (SERS), mass spectroscopy (MS), as well as mass sensitive sensors such as quartz crystal microbalance (QCM), surface acoustic wave (SAW) and mechanical resonators [10,13,14,15,16,17,18]. Resonator-based platforms particularly offer high sensitivity, fast response, accurate and real time measurement, compatibility with integrated circuits and flexibility of readout techniques [18,19,20,21,22,23,24]. The dimensions of cantilevers and strings scale from the micrometer (MEMS) down to the nanometer (NEMS) range [25,26,27,28].

Nanostring biosensors have received increased attention in terms of their sensitivity and amenability to large scale integration [19,29,30,31,32,33]. Such devices assess the addition or loss of bound analytes through monitoring of their resonance frequency. The resonant frequency shift is proportional to the ratio of the mass of the bound molecule to the mass of the nanostring. Smaller string mass therefore improves the sensitivity to captured analytes. Shrinking the size, especially the width, of the string is an effective approach for lowering the mass without altering the physical and mechanical parameters of the sensor system. 

Multiplexed analysis can be accomplished with sensor arrays by employing different molecular probes on devices of different regions, physically isolating the sub-regions, and/or taking advantage of different working modes of the devices [12,24,34,35,36]. Nanostring resonators can readily be scaled up to large arrays featuring up to one million devices per square centimetre [28]. High-density multiplexed assays could be accomplished by dividing such an array into sub-arrays with each sub-array targeting a specific analyte. Lack of reliable linker chemistry however impairs the deployment of glassy nanostrings in such concepts [37,38]. Such a linking layer should offer strong adhesion, biocompatibility, protection against corrosion, long term stability *etc.* [38,39]. Surface functionalization through electrochemical bonding offers strong adhesion and durability compared to weak and fragile physical adsorption [40,41]. However, these chemical processes usually require complex apparatus and are restricted to certain types of surface. For example, electrochemical surface modification requires special tools and is only applicable to conductive and semiconductor materials. Additionally, the process often involves harsh chemicals and processes that deteriorate the material surface or denature the biological compatibility of the sensor system [40,41]. Hence, a simple, milder, versatile and biocompatible linker chemistry is needed.

Diazonium salt reduction-induced aryl film grafting process meets such requirements [40,41,42]. This one-step diazonium salt redox process is readily implemented in aqueous environments at room temperature and ambient pressure and does not require sophisticated equipment. The relatively mild nature of the chemical process preserves the nature of both the modified material and the grafted thin film. Significant research involving diazonium-induced surface modification of biosensors has been conducted in recent years. Most of these reports were however limited to electrochemical sensors and surfaces such as carbon and metals [39,42,43,44,45,46,47,48,49].To the best of our knowledge, diazonium salt linker chemistry has neither been applied to glassy sensor surfaces nor exploited to functionalize nanostrings of the type described here.

The use of diazonium salt modification as linker chemistry for the bio-functionalization of glassy nanostring resonators is reported here. First, SiCN nanostring arrays were fabricated with string widths ranging from 300 nm down to 10 nm using electron beam lithography (EBL). The use of a glassy material is amenable to release through anisotropic etching of the underlying silicon. Such a feature allows strings as narrow as 8 nm, as long as tens of microns, devoid of any undercut, and dried without the need of a critical point drying step [27,50,51]. In addition, SiCN offers tunability of its tensile stress through a controlled post-deposition anneal [52]. Such a feature is not available in other glasses such as SiO_2_ and Si_3_N_4_.

Diazonium-salt induced aryl film modification was then developed on these SiCN surfaces, and analyzed by X-ray photoelectron spectroscopy (XPS). The process was then used to functionalize SiCN nanostring resonators. Anti-rabbit IgG proteins were covalently immobilized onto their surfaces using a common bio-conjugation technique. A blocking layer was then added to inhibit non-specific binding. The nanostrings were then exposed to solutions containing the target analyte, *i.e.*, rabbit IgG. The added mass of the recognition agent and target protein were individually calculated from the resonance frequency shifts. Negative control experiments were performed by exposing similarly functionalized devices to solutions containing goat IgG. Helium ion microscopy (HIM) was conducted on the functionalized and pristine nanostrings to further observe the immobilized analytes.

## 2. Experimental

### 2.1. Fabrication of SiCN Nanostrings

**Figure 1:**
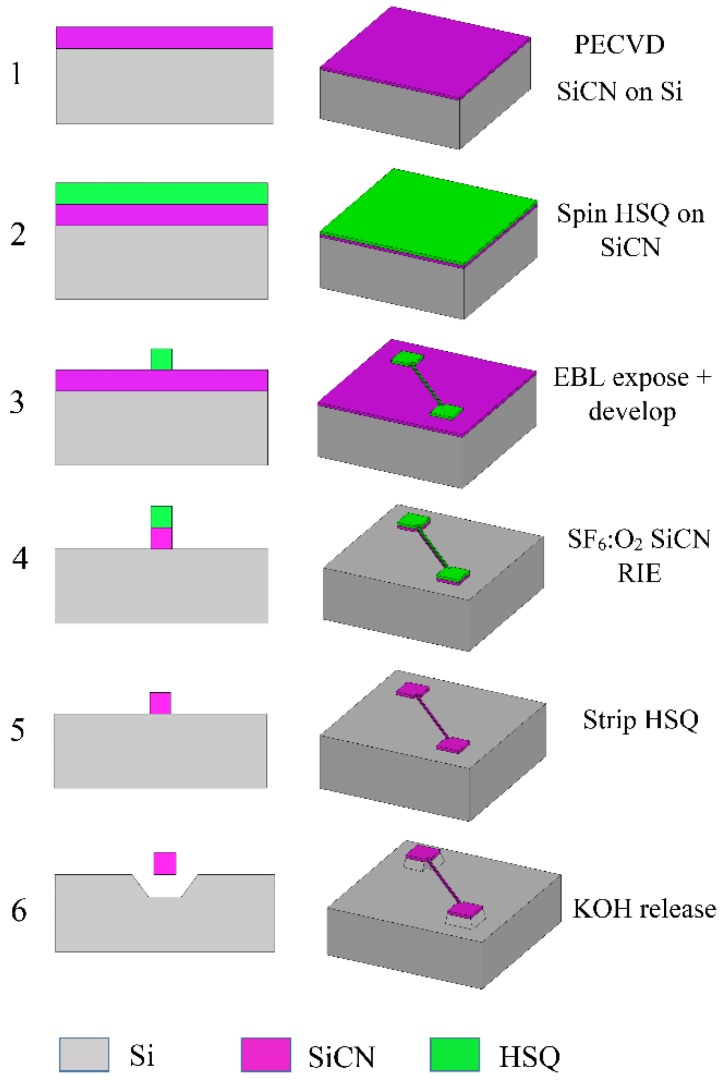
Process flow for the fabrication of SiCN nanostrings.

Figure 1 shows the process flow employed to fabricate the SiCN nanostrings. A single crystal (100) silicon wafer (500 µm thick, 100 mm diameter) was first treated with piranha cleaning solution (3:1 H_2_SO_4_: H_2_O_2_) for 15 min and buffered oxide etch (BOE, 10:1 HF: NH_4_F) for 3 min. A 50 nm thick SiCN layer was deposited by using plasma enhanced chemical vapor deposition (PECVD) at a gas ratio of 4:1 NH_3_: diethylsilane (DES) at a deposition temperature of 300 °C and a pressure of 500 mTorr. The thickness of the SiCN film was measured using a Filmetrics F50 thickness mapping system (Filmetrics, San Diego, CA, USA). The deposited SiCN film was characterized by a Flexus 2320 wafer stress measurement system (KLA Tencor, Milpitas, CA, USA) and exhibited a compressive stress of −764 MPa. The wafer was then annealed in a MiniBrute 3 zone tube furnace (Thermco, Lafayette, NJ, USA) at 525 °C for 2 h after which the SiCN film exhibited a tensile stress of +169 MPa. The wafer was diced into 0.7 cm × 0.7 cm square samples and piranha-cleaned again before EBL resist coating. A prior study of these films [51] showed they possess a density ρ = 2200 kg/m^3^. Such value will thus be used in subsequent analysis.

The SiCN film was patterned using a Raith 150 *^Tw^°* EBL system (Raith Nanofabrication, Dortmund, Germant). Hydrogen silsesquioxane (HSQ, XR-1541) was selected as an EBL resist for its high resolution and simplified process sequence. Diluted 1% HSQ was spun coated onto the SiCN-coated wafer at a spin speed of 4000 rpm for 40 s and then baked at 90 °C for 5 min. The thickness of the HSQ layer was measured to be 28 nm using the Filmetrics system. The sample was exposed in the Raith tool using an electron beam voltage of 10 kV and a 10 µm aperture. For resonator anchoring pads and strings wider than 50 nm, area doses of 0.5–1.75 mC/cm^2^ were used; whereas for strings narrower than 50 nm, line doses of 4.9–9.1 nC/cm were used. After exposure, the HSQ was developed at room temperature in 25% tetramethylammonium hydroxide (TMAH) for 75 s followed by a water rinse and nitrogen dry. With the developed HSQ serving as a protection mask, the SiCN film was selectively etched by reactive ion etching (RIE) using 4:1 SF_6_:O_2_. The HSQ protection layer was then stripped by a 30 s dip in BOE. Finally, the SiCN stripes were released as doubly clamped suspended strings by Si anisotropic etch in 35% KOH solution saturated with IPA at 75 °C for 40–135 s, depending on the string length. Using this process, large arrays of nanostrings with thicknesses of 50 nm, string lengths ranging from 5 to 15 µm, and widths varying from 10 to 300 nm were fabricated.

The resonator arrays were respectively imaged by using a JEOL 6301F SEM (, JEOL, Tokyo, Japan and Hitachi S4800, (Hitachi, Tokyo, Japan) cold field emission SEM) under a low accelerating voltage of 5 kV and ORION NanoFab HIM (,Zeiss, Oberkochen, Germany) under a beam energy of 31 kV and a beam current of 0.4 pA. 

### 2.2. Laser Interferometry Measurement

An optical interferometric technique [53,54] was employed to measure the resonance frequency of the nanostrings. The resonator chip was attached to a piezoelectric disc and located in a vacuum chamber (10^−3^ Torr). The piezoelectric element was actuated using the tracking output of a spectrum analyzer (E4411B, Agilent, Santa Clara, CA, USA). The beam of a 633 nm He-Ne laser source was expanded, power attenuated, directed by a 50: 50 beam splitter and focused by a microscope objective lens on the resonator string surface. The respective reflections from the vibrating string and the underlying substrate induce interferometric modulation of the optical signal. The resulting fringe pattern was passed through the objective lens, redirected by the beam splitter, focused by a convex lens, and impinged on the AC-coupled photo-detector. The photodetector output is then fed back to the spectrum analyzer. The resonant frequency was measured at the largest amplitude of vibration. The resonant frequencies were assessed on the pristine devices, as well as immediately prior and following exposure to the target analyte.

### 2.3. Synthesis of Aryl Diazonium Salt

Diazonium salts were prepared from the corresponding anilines using a previously published method [55]. Briefly, the appropriate aniline (4-bromoaniline or 4-aminobenzoic acid), 0.1 moles, (Sigma-Aldrich, St. Louis, MO, USA) was dissolved in fluoroboric acid (48%, 50 mL, Sigma-Aldrich) and then cooled in an ice water bath. After cooling to 0 °C, sodium nitrite (10 g, Sigma-Aldrich) dissolved in DI water (20 mL) was added drop by drop while stirring. The reaction mixture was further cooled in an ice water bath and stirred for another 1 h. The resultant precipitate was filtered in a Buchner funnel and washed with cold anhydrous ether (Sigma-Aldrich). 

### 2.4. Diazonium Induced SiCN Surface Modification

We employed the diazonium salt induced polymer grafting chemistry process as reported in [41] to modify the SiCN nanostrings. This surface modification process was performed in an aqueous environment at atmospheric pressure and at room temperature. The surface chemistry was first validated on SiCN thin films. Those modified SiCN surfaces were analyzed by XPS to verify the bonding between the grafted layer and the SiCN substrate material.

The diazonium salt 4-bromobenzenediazonium tetrafluoroborate (Br-C_6_H_4_-N_2_BF_4_) was selected as the reactant whereas Br served as a marker element for easy subsequent XPS assessment. L-Ascorbic acid (VC) was selected as the reducing agent. Two identical SiCN bare samples were cleaned in cold (<40 °C) piranha solution (3:1 96% H_2_SO_4_:30% H_2_O_2_) for 15 min and buffered oxide etch (BOE, 10:1 HF:NH_4_F) for 3 min to eliminate organic contamination and possible oxidation on the SiCN surface. A 0.05M 4-bromobenzenediazonium tetrafluoroborate solution and a 0.05 M L-ascorbic acid solution in Milli-Q water were individually prepared. A 2 mL 4-bromobenzenediazonium tetrafluoroborate solution was poured dropwise onto one SiCN chip. Further, 1 mL of L-ascorbic acid (VC) solution was added dropwise to the diazonium solution. The SiCN chips were left to incubate in the mixture in a glass Petri dish for 60 min at room temperature. The sample was then rinsed in water, ethanol, acetone, sonicated in dimethyl formamide (DMF) for 5 min, and dried under nitrogen flow

A negative control experiment was also carried out by similarly immersing chips in a 0.05 M L-ascorbic acid (VC) solution, without any 4-bromobenzenediazonium tetrafluoroborate, for 60 min. 

### 2.5. XPS

The XPS measurements were performed on an AXIS ULTRA spectrometer (Kratos Analytical Ltd, Manchester, country.K.) using a monochromatic Al Kα source (h*ν*=1486.6 eV) at a power of 170 W and 90° take-off angle (TOA). The data was collected from an analysis area of 400 µm × 700 µm. The instrument base pressure was lower than 5 × 10^−8^ Pa. The resolution was 0.55 eV for Ag 3*d* and 0.70 eV for Au 4*f* peaks. The survey scans and the high-resolution spectra were carried out with a pass energy of 160 eV and 20 eV, respectively. For Br, 50× high resolution scans were run with a step of 0.1 eV. An electron flood gun was used for charge neutralization. Data were calibrated by setting the main C 1s component at 284.8 eV. Vision-2 instrument software was employed for data acquisition and CasaXPS was used for its processing. 

### 2.6. Resonator Surface Biofunctionalization

**Figure 2:**
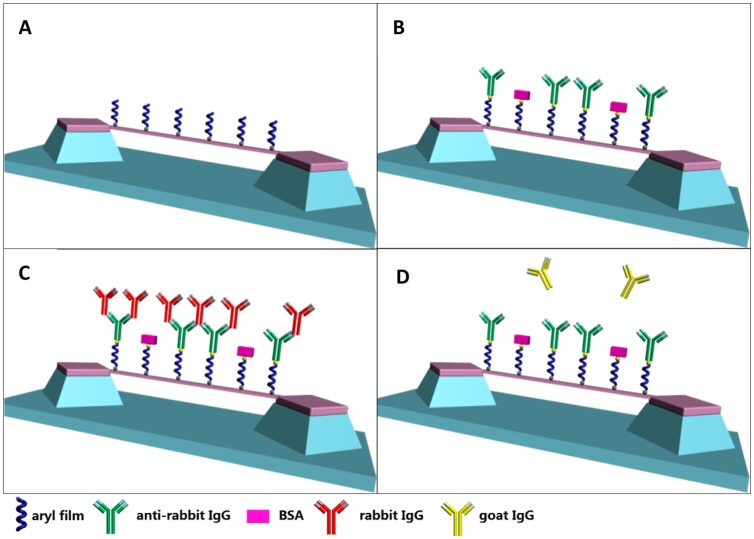
Surface modification and immobilization of molecular probe and target onto SiCN nanostrings. (**A**) SiCN resonators were modified by aryl diazonium salt; (**B**) The probe anti-rabbit IgG antibody was immobilized onto the diazonium layer; (**C**) The target rabbit IgG binds to the probe rabbit IgG antibody; (**D**) The control goat IgG does not bind to the probe rabbit IgG antibody.

The biofunctionalization of the nanostrings, immobilization of molecular probe, and adsorption of target are described in Figure 2.

#### 2.6.1. Resonator Surface Biofunctionalization

First, the SiCN resonators were biofunctionalized using 4-carboxybenzenediazonium tetrafluoro-borate (C_7_H_5_BF_4_N_2_O_2_) as a reactant. As opposed to 4-bromobenzenediazonium tetrafluoroborate, 4-carboxybenzenediazonium tetrafluoroborate supplies an active carboxylic acid group as a chemical active group to bind to the proteins. The procedure was similar to the one described in Section 2.4. Three SiCN resonator chips (A, B, C) were pre-cleaned with the same procedure and incubated in the mixture of 0.05 M 4-carboxybenzenediazonium tetrafluoroborate and 0.05 M L-ascorbic acid, with a 2:1 volume ratio, for 60 min. The chips were then rinsed in water, ethanol, acetone and DMF without sonication to prevent mechanical damage to resonator strings. The aromatic polymer layer with carboxyl group was then grafted onto the nanostring surfaces. 

#### 2.6.2. Activation of Carboxyl Groups

Fresh 0.4 M *N*-(3-dimethylaminopropyl)-*N*′-ethylcarbodiimide hydrochloride (EDC) solution and 0.1 M *N*-hydroxysuccinimide (NHS) solutions were made and mixed together in a 1:1 volume ratio. The carboxyl-bearing SiCN resonator chips were incubated in the mixture for 30 min at room temperature. After this reaction time, the chips were rinsed by phosphate buffered saline (PBS) and blow-dried in nitrogen.

#### 2.6.3. Immobilization of Recognition Bioreceptor

We selected anti-rabbit IgG as bioreceptor of the biosensor and rabbit IgG as target due their high mutual specificity and affinity. Three resonator chips were individually immersed in a solution of 1 mL goat anti-rabbit IgG (100 µg/mL in PBS, polyclonal, Sigma-Aldrich) and incubated at room temperature for 2 h. The goat anti-rabbit IgG was immobilized onto the SiCN resonator surface by covalently binding to the activated carboxyl groups. The chips were rinsed and then immersed in bovine serum albumin (BSA‎, 1% in PBS) at room temperature to block the non-specific binding sites of the surface. After 1 h, the chip surfaces were rinsed and dried.

One resonator chip (A) was loaded in the interferometry system to measure the resonance frequency associated with the added mass of anti-rabbit IgG and BSA. The other two samples (B and C) were employed for the capture of the target protein and for the negative control experiments, respectively.

#### 2.6.4. Capture of Target and Negative Control

The target protein rabbit IgG was adsorbed to the sensor surface by incubating the bioreceptor-grafted resonator chip B in 1 mL of rabbit IgG solution (200 µg/mL in PBS, polyclonal, Sigma-Aldrich) for 1 h at room temperature. This chip was rinsed and nitrogen blow dried. The shift of resonant frequency due to the total mass of anti-rabbit IgG, BSA and rabbit IgG was measured by the optical interferometry system. As a control, chip C was incubated in 1 mL goat IgG solution (200 µg/mL in PBS, polyclonal, Sigma-Aldrich) for 1 h at room temperature, rinsed and dried, and also subjected to resonant frequency measurement.

### 2.7. Helium Ion Microscopy of Functionalized Nanoresonators

High resolution HIM (Zeiss ORION NanoFab) was performed to image the surface protein coverage of the functionalized resonator chip B and a pristine chip as control. Both chips were inspected at beam energy of 31 kV, beam current of 0.4 pA and stage tilt angle of 45°.

## 3. Results and Discussion

### 3.1. SiCN Nanomechanical Resonator Array

**Figure 3:**
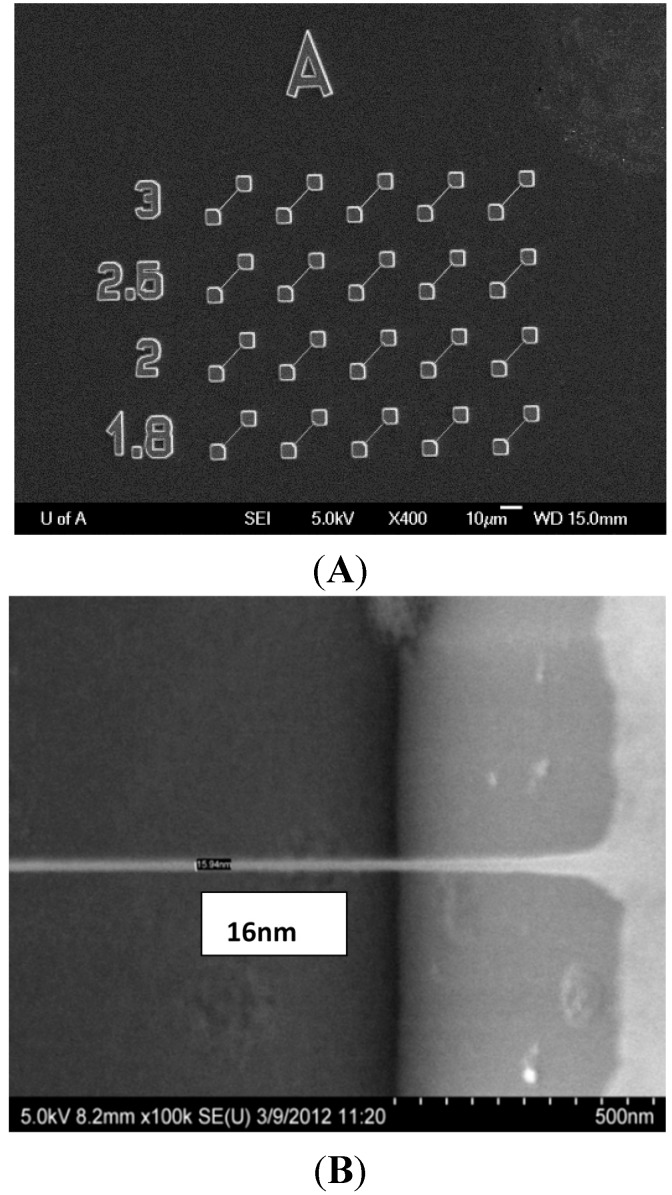
SEM images of SiCN resonators. (**A**) Array of SiCN nanostrings of 15 µm length and varying widths ranging from 180nm to 300 nm; (**B**) A high magnification view of a 16 nm wide string.

A SEM image of a SiCN resonator array with string length of 15 µm and widths ranging from 180–300 nm is displayed in Figure 3A. 

A high magnification view of a 16 nm wide string is shown in Figure 3B. The fabrication yield approached 100%. The SiCN release process by KOH bulk etching silicon was stiction-free due to the high gap between the SiCN string and the silicon substrate. Hence, a critical point drying (CPD) step usually required in most MEMS/NEMS device fabrication was not needed in this process.

### 3.2. One-Step Modification of SiCN Surface

**Figure 4:**
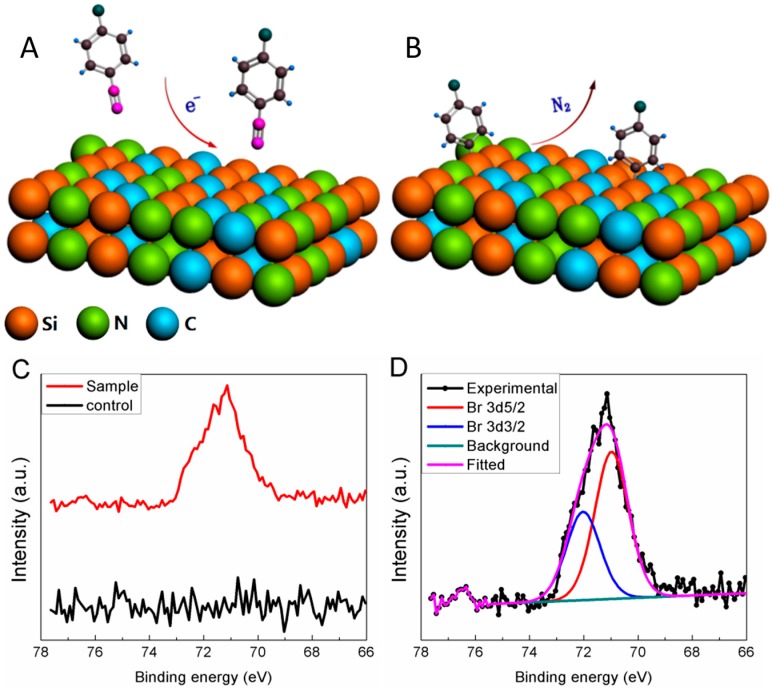
Reaction mechanism and XPS analysis of aryl diazonium modification of SiCN surface: (**A**) Aryl diazonium salt reduction by electrons; (**B**) the created free aryl radicals strongly bond to the SiCN surface; (**C**) XPS high resolution scans of Br element spectra on aryl diazonium modified SiCN sample and control surfaces; (**D**) XPS experimental bromine spectrum and theoretical Br 3d fitted curves.

The SiCN surfaces have been modified in one simple step of redox reaction in an aqueous environment, at ambient pressure and room temperature. The mechanism of aryl diazonium salt-induced surface modification is due to the dediazonation caused by one electron reduction and the creation of free aryl radical [40,41,42]. In the case of the SiCN surface modification described here (Figure 4A,B), the aryl diazonium salt is reduced by L-ascorbic acid, and the free aryl radical forms strong chemical bonds to the SiCN surface atoms.

The SiCN sample modified by 4-bromobenzenediazonium tetrafluoroborate and the negative control were surface analysed by XPS. Bromine was used as marker element to indicate if aryl films were successfully grafted on the sample surfaces. Bromine is indeed a component of the aromatic ring but not a component of the pristine samples. The XPS high resolution spectra (Figure 4C,D) exhibited a significant peak at 71 eV which are attributed to the Br 3*d*5/2 signal. The presence of such a Br peak thus confirmed the bonding of the aromatic ring to the substrate. The high resolution spectrum from the negative control sample is below noise level and does not show any peak at this range. The results demonstrate the successful modification of SiCN surfaces by aryl diazonium salt and confirm the formation and grafting of aryl films on the modified SiCN surface.

4-Carboxybenzenediazonium tetrafluoroborate was employed to further biofunctionalize the SiCN sensor surfaces as the carboxyl group (-COOH) is commonly employed for bioconjugation. However, the compositional elements of this group, carbon and oxygen, also exist in the SiCN substrate. For this reason, Br-benzenediazonium salt was first employed as initial surface modification and XPS analysis. Once the process was validated by XPS, the SiCN nanostrings were further bio-functionalized with 4-carboxybenzenediazonium tetrafluoroborate

### 3.3. Covalent Immobilization of Anti-Rabbit IgG to SiCN Resonator

The resonant frequency of a clamped-clamped beam of rectangular cross-section under a tensile stress *σ*, vibrating in the direction perpendicular to its width, is given by [56]:

(1) $$f_{i}=\frac{\text{22.373}}{4\pi }\sqrt{\frac{Et^{2}}{3\rho l^{4}}+\frac{4\sigma }{\text{22.373}\rho l^{2}}}$$

where *l*, *t*, *E*, and *ρ* are the length, thickness, Young’s modulus and density, respectively. A prior study conducted on similarly fabricated nanostrings showed that a tensile stress of 100–150 MPa was sufficient to have the nanostring deemed as operating in a high-stress limit [51]. With an average tensile stress of ~175 MPa, the nanostrings reported here are thus similarly operating in this regime. The second term of equation 1 thus dominates over the first one, and the relationship becomes:

(2) $$f_{i}=\frac{\sqrt{\text{22.373}}}{2\pi }\sqrt{\frac{\sigma }{\rho }}\frac{1}{l}$$

**Figure 5:**
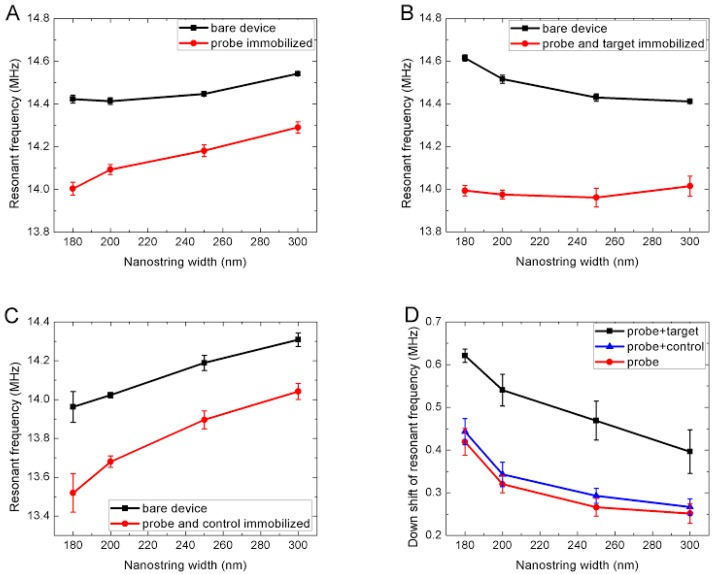
Resonant frequency change of nanostring resonators in samples A, B and C due to surface functionalization. Each of the chips has an array of 20 devices. The resonant frequency is obtained based on statistical analysis of five devices of the same string width. (**A**) Chip A: bare device *versus* the attachment of aryl film, anti-rabbit IgG and BSA. The black squares and red circles respectively designate the bare resonators and resonators immobilized with probe *i.e.*, anti-rabbit IgG and BSA; (**B**) Chip B: bare device *versus* the attachment of aryl film, anti-rabbit IgG, BSA and rabbit IgG. The black squares and red circles respectively designate the bare resonators and resonators immobilized with probe *i.e.*, anti-rabbit IgG and BSA and target rabbit IgG; (**C**) Chip C: bare device *versus* the attachment of aryl film, anti-rabbit IgG, BSA and goat IgG. The black squares and red circles respectively designate the bare resonators and resonators immobilized with probe *i.e.*, anti-rabbit IgG, BSA and control goat IgG; (**D**) Comparison of down shifts of resonant frequencies due to the surface adsorption of probe, probe and target, and probe and control. The red circles designate the resonators of chip A immobilized with probe *i.e.*, anti-rabbit IgG, BSA. The black squares correspond to the resonators of sample B immobilized with probe and target rabbit IgG. The blue triangles designate the resonators of sample C immobilized with probe and control goat IgG.

More specifically, the strings employed in this study possessed a length l = 15 μm and a density of ρ = 2200 kg/m^3^ [51]. The frequency of the first resonance mode is thus expected to be f_i_ = 14.2 MHz. Figure 5 shows that the bare devices displayed resonant frequencies ranging from f = 14.0 MHz to f = 14.6 MHz, thus in agreement with Equation (2). The range of experimental frequencies observed is attributed to local variations of stress within the wafer, as was observed in prior studies [27,51,52].

The carboxyl group introduced onto the SiCN surface enabled the covalent immobilization of proteins by formation of an amide bond between protein and the surface. EDC/NHS was used to activate the carboxyl groups. The carboxylic acid group was converted to carboxyl-NHS ester using EDC as an intermediate. The NHS ester reacts with the primary amine in proteins and forms an amide bond. This strategy was thus employed to covalently bind anti-rabbit IgG onto the nanostrings. In turn, BSA was used to block the non-specific binding sites.

The binding of anti-rabbit IgG and BSA onto the nanostrings was quantified through assessment of resonant frequency downshifts. Figure 5A reports the resonant frequency of the nanostrings before and after the immobilization of anti-rabbit IgG and BSA. 

Significant average frequency downshifts from 251.5 ± 22.8 kHz to 419.4 ± 31.3 kHz, corresponding to different resonator string widths, were observed. The corresponding added mass was calculated using Equation (3):

(3) ∆f=f0∆ms2ρ(1t+1w)

where ${\text{f}}_{0}$ and ∆f are the unloaded resonant frequency and the absolute value of frequency shift induced by the loaded mass, ∆m/s is the mass-per-area; and ρ, s, v, l, t, w are the density, effective area, volume, length, thickness and width of the nanostring, respectively. Table 1 summarizes the added mass inferred from these experiments. An average mass-per-area of 1.94 ± 0.20 $\text{fg}/{\text{µm}}^{2}$ was obtained for different string widths. It was observed that the frequency shift increased as the string width decreases, *i.e.*, narrower strings showed more sensitive response. This can be expected from the following argument. Since ${\text{f}}_{0}$ does not change with string width [51], ∆f is proportional to the ratio of the effective surface area and the volume of the string, assuming that the biomolecules are uniformly distributed on the string surfaces *i.e.* the added mass-per-area remains constant for different string width. Further derivation of Equation 3 indeed shows an increase of ∆f as the string width decreases (Equation (3)).

**Table 1:** Analysis of average resonant frequency shift and added mass of the resonator array of sample A due to the attachment of aryl film, anti-rabbit IgG and BSA.

Nanostring Width (nm)	300	250	200	180
Average unloaded frequency f_0_ (MHz)	14.542 ± 0.0080	14.447 ± 0.0087	14.4135 ± 0.0157	14.4232 ± 0.0184
Average shift frequency due to probe Δf (kHz)	250 ± 23	265 ± 21	320 ± 20	420 ± 31
Added mass of probe Δm (fg)	17 ± 1.56	15 ± 1.2	14.7 ± 0.92	17.3 ± 1.3
Added mass-per-area of probe Δm/s (fg/µm^2^)	1.6 ± 0.15	1 ± 0.13	1.95 ± 0.12	2.50 ± 0.19

### 3.4. Specific Detection of Target Rabbit IgG Attached to Resonator

Figure 5B shows nanostring frequency shifts due to the immobilization of anti-rabbit IgG, BSA and rabbit IgG (sample B). In turn, Table 2 summarizes the total added mass inferred from these shifts. The mass of the target rabbit IgG was obtained by subtracting the combined mass of the anti-rabbit IgG and BSA from the total combined mass of rabbit IgG, anti-rabbit IgG, and BSA. 

**Table 2:** Analysis of average resonant frequency shift and added mass of the resonator array of sample B due to the attachment of aryl film, anti-rabbit IgG, BSA and target rabbit IgG.

Resonator String Width (nm)	300	250	200	180
Average unloaded frequency f_0_ (MHz)	14.411 ± 0.0089	14.4298 ± 0.0175	14.5155 ± 0.0216	14.6142 ± 0.0147
Average shift frequency due to probe and target Δf (kHz)	395 ± 51	470 ± 46	541 ± 37	621 ± 16
Added mass of probe and target Δm (fg)	27 ± 3.5	27 ± 2.6	24.5 ± 1.5	25.3 ± 0.6
Added mass-per-area of probe and target Δm/s (fg/µm^2^)	2.6 ± 0.3	3.0 ± 0.3	3.3 ± 0.22	3.66 ± 0.09
Added mass-per-area of target Δm/s (fg/µm^2^)	0.96 ± 0.29	1.29 ± 0.30	1.42 ± 0.23	1.07 ± 0.21
Total number of target molecules	40,500 ± 1200	46,500 ± 1100	43,000 ± 6800	30,000 ± 5800

An average added mass-per-area of 1.18 ± 0.09 $\text{fg}/{\text{µm}}^{2}$ is thus attributed to the capture of rabbit IgG. Given the IgG molecular weight of 150 kDa, this corresponds to one rabbit IgG molecule per 214 ${\text{nm}}^{2}$. Since one IgG molecule occupies a plane area of about 70 ${\text{nm}}^{2}$ [57], the overall coverage of target rabbit IgG on the resonator surfaces is estimated at roughly 33%.

Negative control experiments were performed to verify the specificity of this detection (sample C). The negative controls consisted of exposing nanostrings similarly functionalized with anti-rabbit IgG to a solution rather containing goat IgG. Figure 5C reports the nanostring frequencies before and after their exposure to anti-rabbit IgG, BSA and goat IgG. The frequency shift due to any attachment of goat IgG was obtained by subtracting the frequency shift associated to the BSA and anti-rabbit IgG probe from the shift associated to bound goat IgG, anti-rabbit IgG, and BSA (Table 3). As expected, the frequency shifts related to bound goat IgG are negligible compared to the frequency shift associated to bound rabbit IgG (Table 2). The average frequency shifts observed in those negative controls are indeed at least one order of magnitude smaller than those observed in the positive capture experiments, indicating minimal non-specific attachment of non-target protein to the nanostrings. Capture of small amounts of goat IgG remain possible given the polyclonal nature of the two targets, and thus the finite cross species reaction of goat IgG with anti-rabbit IgG. Hence, the significantly larger shift obtained from the target protein compared to the non-targeted one demonstrates the potential of this platform for molecular fingerprinting and multiplexed assays involving a large number of devices.

**Table 3:** Analysis of average resonant frequency shift of the resonator array of sample C due to the attachment of aryl film, anti-rabbit IgG, BSA and control goat IgG.

Resonator String Width (nm)	300	250	200	180
Average unloaded frequency (MHz)	14.31 ± 0.035	14.20 ± 0.04	14.0234 ± 0.0140	13.97 ± 0.08
Average shift frequency due to probe and control (kHz)	267 ± 19	295 ± 18	345.0 ± 28	440 ± 31
Average shift frequency due to control (kHz)	15 ± 33	27 ± 24	23 ± 47	24 ± 50
Average shift frequency ratio of control to target (percentage)	10.6%	13.3%	9.7%	12.8%

Given that the native frequencies of bare nanostrings vary slightly within and across chips, the net frequency downshifts for samples A, B and C are plotted for comparison purposes in Figure 5D. As reported through Table 2 and Table 3, the attachment of the probe onto the nanostring causes a significant frequency downshift and the subsequent binding of target leads to a further significant shift. Meanwhile, while the exposure of the negative control results in significantly smaller shifts. In all three cases, nanostrings with the same length but narrower width tend to display larger frequency shifts, as expected from the model derived in Section 3.4. The detection threshold is thus effectively improved by using narrower strings. The chemical processes may themselves have imparted surface stress that influenced resonance. Such phenomena have been thoroughly studied in cantilever sensors [58]. Such an effect is typically modelled as an effective additional tension applied to the string. This being said, the associated frequency shifts are expected to be in the range of δf/f_r_ ~ 10^−4^, where f_r_ is the resonant frequency of the unstressed beam [59]. The nanobeams employed here would possess an unstressed resonant frequency of ~2 MHz. Any effect of surface stress would thus be in the order of a few hundreds of Hertz. This range is a thousand-fold less than the net mass-loading shifts reported here, which are in the tens to hundreds of kilohertz. Evidently, surface stress effects may readily account for some of the experimental errors and noise levels observed in our negative control experiment.

### 3.5. HIM Protein Observation on the Resonator Surface

**Figure 6:**
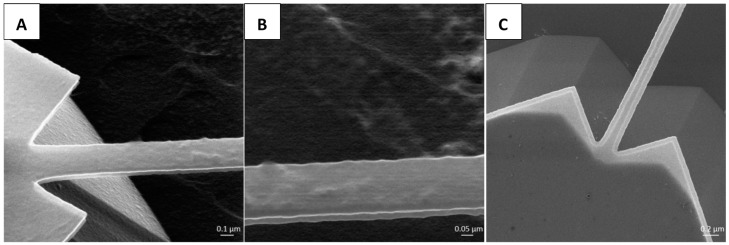
Helium ion micrographs of a SiCN resonator immobilized with anti-rabbit IgG and rabbit IgG with contrast to its pristine surface. Images taken at a 45° tilt angle (**A**) The protein distribution on SiCN nanoresonator anchoring pad and suspended nanostring; (**B**) High magnification view of protein coverage on the suspended nanostring; (**C**) Pristine surface of a SiCN nanoresonator before functionalization.

As seen in the HIM images of Figure 6A,B, a protein layer was wrapped onto the suspending SiCN nanostring and grafted on the SiCN anchoring pad surface of the functionalized nanoresonator chip B. It is noticeable that the protein layer also formed on the Si surface of the slope of the anchoring pad and the substrate. This is to be expected given that diazonium chemistry is also known to functionalize Si surfaces [42]. 

In contrast, Figure 6C shows the surface of a pristine nanostring. Both SiCN nanostrings and Si substrate are markedly smoother and devoid of the lumps associated to polymer attachment. More specifically, the thin undercut slope caused by a previous etching step is observed at the edges of the SiCN nanostring and the anchoring pad. Such observations further support the notion that the lumpy material seen in Figure 6A is related to the biological analytes. 

## 4. Conclusions/Outlook

We have reported the use of diazonium salt-induced surface modification as linker chemistry for the biofunctionalization of glassy nanostring resonators. The chemical bonding between the grafted aryl film layer and the SiCN surface was first verified by XPS. High affinity anti-rabbit IgG and rabbit IgG were immobilized onto similarly modified nanostrings as molecular probe and target, respectively. Bonding of the probe and target was detected through significant shifts of the nanostring resonant frequency. In turn, negative control experiments showed negligible frequency shifts, confirming the specificity of the detection. High resolution HIM further verified the grafting of the proteins on the nanostrings. Using diaznonium modification chemistry offers greater biocompatibility and a more stable chemical bonding for such applications. This method could readily be expanded to multiplexed assays using diazonium salts bearing different bio-conjugation groups and different molecular probes.
